# AI-Based Models for Diabetic Foot Ulcer Assessment: Scoping Review

**DOI:** 10.2196/77925

**Published:** 2026-07-08

**Authors:** Muhamad Zulfiqar, Saldy Yusuf, Muhammad Jufri Taming, Herlina Burhan

**Affiliations:** 1 Faculty of Nursing, Hasanuddin University, Jl. Perintis Kemerdekaan KM 10, Makassar, South Sulawesi, 90245, Indonesia, 62 81241841800; 2 Indonesian Diabetic Foot Care Research Group (IndoFootCare), Hasanuddin University, Makassar, South Sulawesi, Indonesia

**Keywords:** artificial intelligence, deep learning, diabetic foot ulcers, diabetic wound, scoping review

## Abstract

**Background:**

Diabetic foot ulcers (DFU) are serious complications of diabetes that contribute substantially to morbidity, mortality, and health care burden. Accurate and timely wound assessment is essential for effective DFU management; however, conventional assessment methods are limited by subjectivity, time constraints, and interobserver variability.

**Objective:**

This scoping review aimed to map and synthesize evidence regarding the development and application of artificial intelligence (AI)–based models for DFU assessment.

**Methods:**

A scoping review was conducted following the Arksey and O’Malley framework and reported according to the PRISMA-ScR (Preferred Reporting Items for Systematic Reviews and Meta-Analyses Extension for Scoping Reviews) guidelines. Literature searches were performed in PubMed, ProQuest, and Scopus for studies published between 2014 and 2026. Study selection and data charting were conducted independently by two reviewers using predefined inclusion criteria based on the PCC (population, concept, context) framework. Extracted data were synthesized narratively and categorized according to major AI application domains.

**Results:**

A total of 654 records were identified, of which 46 studies met the inclusion criteria. The included studies predominantly focused on image segmentation, diagnostic classification, and risk prediction or monitoring of DFUs. Convolutional neural networks were the most commonly applied models, with performance evaluated using metrics such as accuracy, Dice similarity coefficient, and area under the curve. Most studies relied on retrospective, single-center datasets, with limited external validation and minimal real-world clinical implementation.

**Conclusions:**

AI-based models demonstrate strong potential to enhance DFU assessment and monitoring by improving accuracy and efficiency. However, significant gaps remain in terms of dataset diversity, external validation, and integration into clinical workflows. Future research should prioritize prospective validation, standardized datasets, and real-world implementation to support safe and effective clinical adoption.

## Introduction

Diabetic foot ulcers (DFUs) significantly increase the risk of morbidity and mortality in patients with diabetes. The lifetime incidence of DFU ranges from 19% to 34%, with an annual incidence of 2%. Although DFU can heal, the recurrence rate remains high: at 40% within 1 year and 65% within 3 years [1]. DFUs are associated with significant morbidity and mortality, with a 5-year mortality risk that is 2.5 times higher than that of diabetic patients without DFU [2]. The management of DFU faces significant challenges, including the complexity of their causes, slow healing processes, and high risk of complications, such as infections and amputations [3]. Additionally, patients with DFUs often experience a considerable decline in their quality of life, including chronic pain, mobility limitations, and psychological distress, such as anxiety and depression [4]. Therefore, appropriate DFU management, particularly wound assessment, is crucial for successful treatment.

Wound assessment is the initial step in managing DFU. Accurate and comprehensive wound assessment is critical for wound management [5]. Wound assessment tools provide scores or values that reflect changes in the clinical condition [6]. Several wound assessment tools are available, including the Bates-Jensen Wound Assessment Tools [7], the Diabetic Foot Ulcer Assessment Scale (DFUAS) [8], the DMIST (depth, maceration, inflammation/infection, size, tissue type of the wound bed, type of wound edge, and tunneling/undermining) tool [9], and the DEPA (depth, extent of bacterial colonization, phase of healing, and associated aetiology) tool [10]. Typically, wound assessment and monitoring are performed by nurses with specialized training in wound care [11]. Wound assessment depends on health care providers’ clinical experience, which may lead to variations in diagnosis and treatment [12]. Some studies have shown that insufficient documentation in wound assessment can hinder the early detection of complications and negatively affect treatment outcomes [13]. Furthermore, conventional methods of assessing the severity of DFU are often time-consuming, labor-intensive, and prone to discrepancies, which hampers accurate patient monitoring [14]. Another challenge is the technological limitations in wound assessment, as manual wound documentation is often inaccurate and can lead to inconsistencies in care [15]. Therefore, innovation is needed to enhance the efficiency and objectivity of wound assessment.

The field of artificial intelligence (AI) is rapidly expanding, particularly in health care. AI can be applied to diagnose diseases, design personalized treatment plans, and assist clinicians in decision-making [16]. With technological advancements, several commercially available wound assessment or monitoring systems are now available to track chronic wounds [17]. AI can help improve wound assessment accuracy, patient engagement, and compliance with wound care regimens [18]. AI has the potential to significantly enhance the early detection and management of DFU.

Accurate and objective assessment of DFU remains challenging in clinical practice. Recently, AI approaches using digital image analysis have been increasingly explored to support DFU screening and evaluation. Previous systematic reviews have reported that convolutional neural network (CNN)–based models are the most commonly used approaches for DFU image segmentation and screening [19]. Furthermore, AI has proven to be more accurate than conventional methods for wound image analysis [20]. The advancement of AI technology allows for the development of more accurate and efficient wound assessment models with deep learning algorithms that achieve accuracy comparable to that of nurses in diagnosing DFU. These systems provide reliable assessments to formulate personalized care plans, aligning with research showing machine learning algorithms’ potential in DFU recognition [21]. AI has demonstrated promising results in the classification and localization of DFU, with high accuracy in identifying ischemia and infection, ranging from 73% to 95.4% [22]. Wound assessment systems, ranging from computer-based to mobile applications, have the potential to integrate AI to enhance measurement accuracy [23]. Despite several commercially available wound assessment systems, most of these systems have not been reviewed in the literature regarding measurement accuracy, mainly since DFU may occur in curved or angled areas on the foot [17]. Therefore, this scoping review aimed to map and synthesize evidence regarding the development and application of AI-based models for DFU assessment.

## Methods

### Scoping Review Framework

This scoping review was conducted in accordance with the 5-stage framework proposed by Arksey and O’Malley [24] and reported according to the PRISMA-ScR (Preferred Reporting Items for Systematic Reviews and Meta-Analyses Extension for Scoping Reviews) guidelines [25]. This review aimed to map and synthesize evidence regarding the development and application of AI-based models for DFU assessment.

The scoping review methodology enables systematic mapping of the existing literature, identification of research gaps, and synthesis of available evidence. The methodological framework consisted of five stages: (1) identifying the research question, (2) identifying relevant studies, (3) study selection, (4) data charting, and (5) collating, summarizing, and reporting the results.

### Stage I: Research Question

The research question guiding this review was: How have AI-based models been developed and applied for diabetic foot ulcer assessment, and what are their methodological characteristics and reported-performance outcomes?

### Stage II: Identifying Relevant Studies

A comprehensive literature search was conducted in PubMed, ProQuest, and Scopus to identify relevant studies published between 2014 and 2026. The search strategy included combinations of keywords related to diabetic foot ulcers, artificial intelligence, machine learning, deep learning, and wound assessment. The detailed search strings for each database are provided in Multimedia Appendix 1. The eligibility criteria were defined using the PCC (population, concept, context) framework recommended by the Joanna Briggs Institute.

*Population* included studies involving individuals with DFU, without restriction on age, sex, or disease severity. *Concept* focused on studies describing the development or application of AI-based models, including machine learning, deep learning, and computer vision approaches, for DFU assessment, encompassing tasks such as segmentation, classification, risk prediction, monitoring, and decision support. *Context* included studies conducted in clinical, experimental, or health care–related technological settings, without geographical restriction.

Only peer-reviewed full-text articles published in English between 2014 and 2026 were included. Efforts were made to obtain full-text articles through institutional access and other available sources to minimize the risk of exclusion based on access limitations.

### Stage III: Study Selection

All retrieved records were imported into Rayyan for management and screening purposes. Duplicate references were identified and removed prior to review [26]. Title and abstract screenings were conducted independently by two reviewers. Studies deemed potentially eligible proceeded to independent full-text assessment by the same reviewer. Disagreements at any stage of the screening process were resolved through discussion for agreement. When agreement was not reached, a third reviewer was consulted.

A total of 654 records were identified through database searches, including PubMed (n=245), ProQuest (n=123), and Scopus (n=286). After removing 310 duplicate records, 344 records remained and were screened based on titles and abstracts, of which 196 were excluded. Subsequently, 148 reports were sought for retrieval. Of these, 41 reports could not be retrieved after attempts to access full texts through available institutional and alternative sources. As a result, 107 full-text articles were assessed for eligibility. Following full-text assessment, 61 articles were excluded because they were not related to DFU. Ultimately, a total of 46 studies were included in this review.

### Stage IV: Data Mapping

Data were extracted to capture study characteristics, including author and year, country, study design, dataset characteristics, AI methods, application domain, and reported performance metrics. Information regarding the application of AI-based assessment models for DFU was also extracted.

### Stage V: Thematic Summary and Key Findings

The extracted data were synthesized narratively using a deductive thematic approach. This approach was guided by predefined analytical domains developed based on the review objective and commonly reported categories of AI applications in DFU assessment. The predefined domains included segmentation and measurement, diagnostic classification, risk prediction and monitoring, and clinical decision support systems. Two reviewers independently reviewed the extracted data and assigned each study to one or more of the predefined domains based on its primary application focus. During this process, studies were examined for their methodological characteristics, application objectives, and reported outcomes to ensure accurate categorization. Any discrepancies in domain classification were resolved through discussion to reach consensus. When necessary, a third reviewer was consulted to adjudicate disagreements. This process ensured consistency and reliability in the thematic grouping of the included studies. Consistent with the scoping review methodology, no formal risk-of-bias assessment was conducted.

### Ethical Considerations

This scoping review was not prospectively registered in any database. As this study synthesized data from publicly available literature and did not involve human participants or identifiable data, formal ethical approval was not required.

## Results

### Study Characteristics

**Figure 1.**
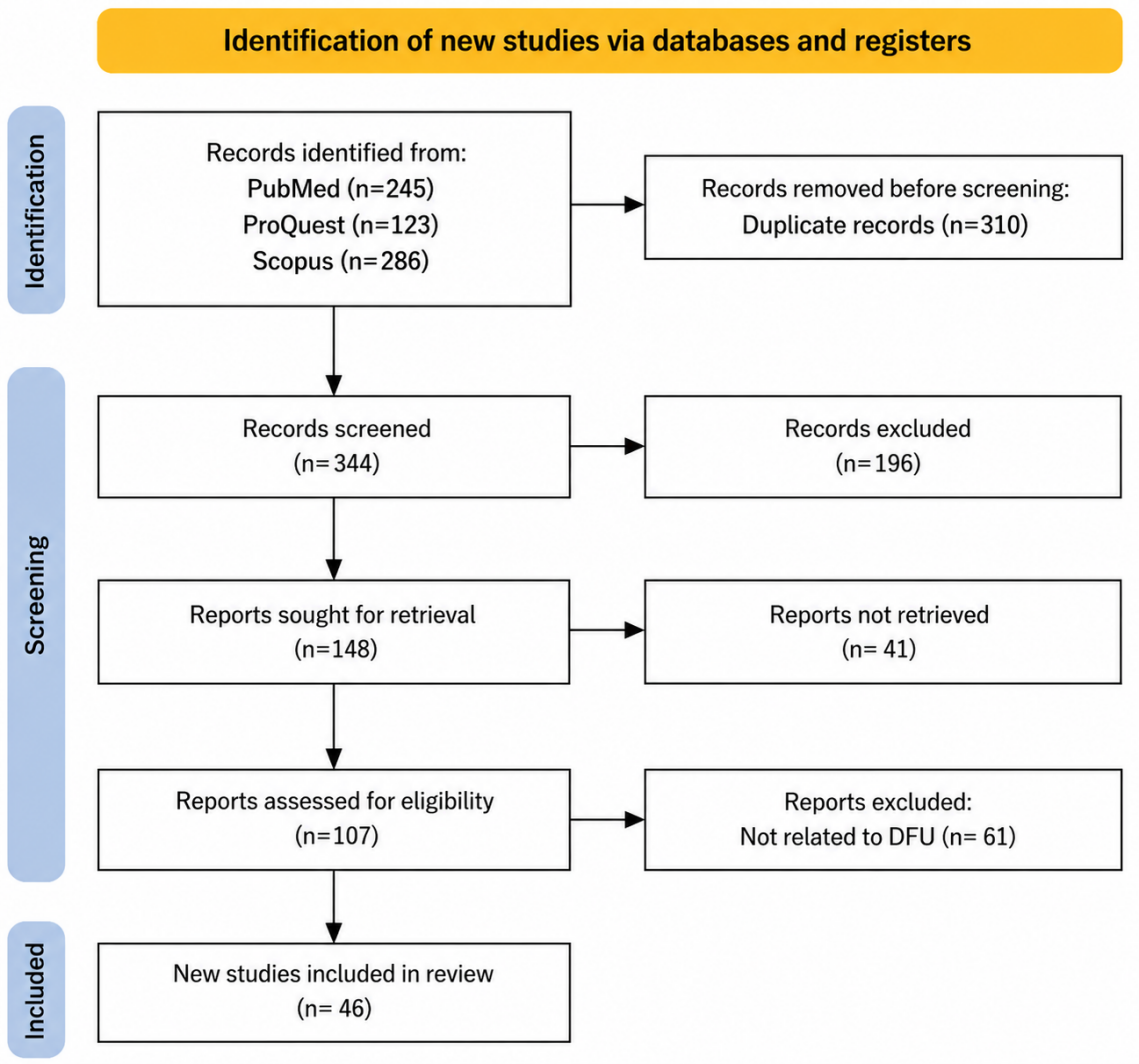
Flow diagram of study search and selection based on PRISMA-ScR. DFU: diabetic foot ulcer.

A total of 46 studies were included in this review (Figure 1). The key characteristics of the included studies, including wound assessment task, AI methods, data sources, and primary outcomes (Table 1).

**Table 1.** Overview of artificial intelligence (AI) methods and application domains in the included studies.

Characteristics	Studies	AI methods^a^	Data source^b^	Primary outcome
Wound segmentation, measurement, and characterization	[17,27-38]	Fast R-CNN; bi-CNN; DL-based mobile app; SwishRes-U-Net; wound viewer AI; AHRF; AI measurement app; CNN; explainable DL; CNN-based detection; thermal DL; benchmark DL; DL segmentation	DFU image datasets; DFU photos; DFU/FUSEG datasets; clinical patients; multidevice images; public datasets; multi-dataset DFU	Wound localization; depth and granulation measurement; automated wound measurement; tissue boundary extraction; accurate 3D measurement and wound bed preparation classification; edge detection; measurement consistency; accurate wound segmentation and classification; interpretable segmentation; granulation detection; improved detection accuracy; standardized evaluation; generalizability analysis
Diagnostic classification and condition assessment	[14,39-52]	Hybrid DL classifier; FusionNet (XAI); ScoreDFUNet; DenseVAE-CL; ConMatFormer; few-shot DL; multimodal DL; attention DL; hybrid CNN; CNN+Transformer; efficient CNN; lightweight CNN; explainable DL; Transformer+XAI; CNN+ELM	DFU images; 2673 Kaggle images; DFUC2021; Kaggle datasets; clinical images	Condition classification; explainable diagnosis; image-based diagnosis; very high classification accuracy; improved accuracy and explainability; accurate classification in limited datasets; improved classification performance; enhanced feature extraction; efficient and interpretable classification
Risk prediction, complication detection, and longitudinal monitoring	[22,53-65]	LightGBM; AutoML DL; DFUCare platform; DFU-Helper; CNN+SPCD; Siamese neural network; Mask R-CNN thermal fusion; ML model; temporal ML; AI monitoring system; deep learning	Clinical datasets; DFU datasets; clinical images; serial DFU images; prospective clinical patients; population datasets; longitudinal data	Outcome prediction; infection and ischemia detection; healing trend monitoring; status differentiation; area measurement and healing monitoring; amputation prediction; healing prediction; diabetic foot ulcer occurrence prediction; healing trajectory prediction; severity monitoring; early complication detection
Clinical decision support and automated model development	[44,66-68]	AI-based DSS; AutoML DL; ML decision model; explainable DL	DFU clinical cases; DFU datasets; clinical datasets	Decision agreement; automated model generation; risk stratification; transparent clinical decision-making

a AHRF: adaptive hybrid regression framework; AI: artificial intelligence; AutoML: automated machine learning; bi-CNN: bilinear convolutional neural network; CNN: convolutional neural network; DL: deep learning; DSS: decision support system; ELM: extreme learning machine; LightGBM: light gradient boosting machine; ML: machine learning; R-CNN: region-based convolutional neural network; SPCD: superpixel color descriptor; XAI: explainable artificial intelligence.

b DFU: diabetic foot ulcer; DFUC: Diabetic Foot Ulcer Challenge; FUSEG: Foot Ulcer Segmentation Dataset.

Diagnostic classification and condition assessment (n=15, 32.6%) and risk prediction, complication detection, and longitudinal monitoring (n=14, 30.4%) were the most frequently investigated domains, followed by segmentation, measurement, and characterization (n=13, 28.3%). Clinical decision support and automated model development were less frequently studied (n=4, 8.7%) (Table 2). This distribution reflects a strong research emphasis on image-based analysis and predictive modeling, with comparatively limited exploration of clinically integrated decision-support systems.

To further examine the distribution of evidence and identify potential research gaps, an evidence gap map was developed based on AI application domains and key methodological characteristics (Table 3).

**Table 2.** Distribution of artificial intelligence application domains in included studies (N=46).

Artificial intelligence application domain	Studies, n (%)
Diagnostic classification and condition assessment	15 (32.6)
Risk prediction, complication detection, and longitudinal monitoring	14 (30.4)
Segmentation, measurement, and characterization	13 (28.3)
Clinical decision support and automated model development	4 (8.7)

**Table 3.** Evidence gap map of artificial intelligence (AI) application domains in diabetic foot ulcer assessment.

Characteristics	Segmentation, measurement, and characterization	Diagnostic classification and condition assessment	Risk prediction, complication detection, and longitudinal monitoring	Clinical decision support and automated model development
Image-based diagnosis	✓	✓	✓	✓
Clinical validation datasets	X	✓	✓	✓
Prospective studies	X	X	X	X
External validation	X	X	✓	X
Real-world implementation	X	X	✓	X
Explainable AI (XAI)	✓	✓	✓	✓
Multimodal data	✓	✓	X	X

The evidence gap map illustrates the distribution of studies across AI application domains and key methodological characteristics. The findings demonstrate a strong concentration of evidence in segmentation and diagnostic classification domains, particularly those using image-based datasets, indicating a dominant reliance on computer vision approaches for DFU assessment. In contrast, the use of clinical or tabular data remains limited and is primarily observed in studies focusing on risk prediction and monitoring. Notably, prospective study designs were absent across all domains, and external validation was rarely reported, with only limited evidence identified in prediction-related studies. Furthermore, real-world implementation of AI models remains scarce, with only a small number of monitoring studies demonstrating initial clinical application. Although explainable AI approaches have been increasingly incorporated across domains, their integration remains inconsistent. Overall, these findings highlight that while the technical development of AI models for DFU assessment is rapidly advancing, substantial gaps persist in clinical validation, generalizability, and real-world implementation.

This scoping review mapped the application of AI-based assessment models in DFU. Various studies have been conducted to enhance the detection, evaluation, and monitoring of DFU using AI, machine learning, and computer vision approaches. Some studies have focused on predicting amputation levels in DFU patients by using explainable machine learning models that classify DFU based on the Wagner and WIfI systems while also evaluating risk factors such as the duration of diabetes, history of amputations, and vascular conditions [53]. Additionally, machine learning–based models have been used to identify risk factors for DFU through local foot examination [54]. In wound detection and segmentation, several studies have developed deep learning–based models, such as Fast R-CNN, which enable automatic measurement of wound size, boundaries, and geometry of DFU [27]. A bilinear CNN-based model was also developed for fine-grained wound classification based on depth and granulation tissue [28]. Meanwhile, another study explored explainable AI approaches using algorithms such as SHAP (Shapley Additive Explanations), LIME (local interpretable model-agnostic explanations), and Grad-CAM (gradient-weighted class activation mapping), which enhance transparency in DFU detection and classification [39]. New datasets have also been developed to identify ischemia and infection in DFU using computer vision techniques [55].

Smart application–based technology has been integrated into DFU monitoring through various innovative applications, enabling real-time wound dimension measurement, validation with manual methods, and automatic tissue classification [29]. Furthermore, hardware acceleration approaches such as field-programmable gate array (FPGA) and graphics processing unit (GPU) have been developed to enhance the efficiency of real-time wound classification [40].

Regarding wound boundary determination, the associative hierarchical random field (AHRF) method was introduced as a more accurate alternative to traditional methods by reducing dependence on lighting and camera angles [30]. Another study developed an AI-based automated scoring system to assess DFU severity by considering wound classification, size, and color characteristics [14]. AI-based approaches have also been used to assess amputation levels in DFU patients based on clinical photographs, compared to physician decisions using Wagner classification [66]. Deep learning–based platform development has also been conducted for noninvasive DFU detection and monitoring [22]. The SwishRes-U-Net model has been applied to improve DFU evaluation accuracy [31]. Meanwhile, a Siamese neural network–based framework has been developed for longitudinal DFU evaluation to monitor wound progression under various clinical conditions [56].

Several studies have focused on quantitative wound assessment using advanced imaging technologies. Similarly, an AI-powered medical device (Wound Viewer) has been validated for remote wound assessment, showing strong agreement with clinician evaluations in measuring wound parameters and classifying tissue characteristics [32].

Other studies have primarily focused on image segmentation and severity classification. An AI-enhanced imaging framework combining active contour modeling and deep learning classification has been developed to segment ulcer boundaries and predict severity based on Wagner grading, achieving high segmentation accuracy [57]. In addition, a Mask R-CNN model integrated with thermal and visual image fusion has been applied to evaluate DFU healing trajectories, demonstrating a strong correlation with clinician-based measurements [58].

Several deep learning architectures have been proposed for diagnostic classification. A hybrid deep learning architecture integrating convolutional, attention, and transformer modules has been introduced for DFU classification, reporting high accuracy and improved interpretability through explainable AI methods [67]. Another framework combining DenseNet, variational autoencoders, and contrastive learning was developed to improve the classification performance and model generalization [41]. Additionally, an ensemble deep learning system combining YOLOv8 and Faster R-CNN was developed to enhance DFU detection and localization accuracy, demonstrating improved performance compared with individual models [42].

Automated DFU detection and classification using traditional machine learning approaches has been demonstrated as feasible in early image-based studies [69], providing a foundation for subsequent methodological advancements. Building on this, deep learning–based architectures have further improved classification performance by enabling more robust feature extraction from DFU images [43]. More recently, transformer-based models have enhanced classification accuracy while improving feature representation and generalization across datasets [44], and hybrid architectures combining CNNs and vision transformers have further strengthened DFU detection performance by integrating local and global feature learning [45]. In data-limited scenarios, few-shot learning approaches have enabled effective DFU classification despite constrained labeled datasets [46], while attention-based deep learning models have improved feature extraction and classification accuracy by focusing on clinically relevant regions of the image [47].

Beyond performance improvements, explainable AI methods have enhanced transparency in DFU detection by providing visual explanations of model predictions [48], thereby supporting clinical interpretability. At the same time, lightweight deep learning architectures have demonstrated high performance in DFU detection and grading tasks with reduced computational burden, facilitating real-time applications [49]. Multimodal deep learning approaches integrating RGB (red, green, and blue) and thermal data have further improved classification accuracy by incorporating complementary physiological information [50], while texture-based feature extraction combined with deep learning has also been shown to enhance classification performance [51].

In parallel with classification advancements, deep learning–based segmentation approaches have been widely used to delineate wound boundaries and support quantitative wound assessment [33]. However, cross-dataset evaluation studies have highlighted ongoing challenges in model generalizability, particularly when applied to heterogeneous clinical data [34]. To address this, thermography-based segmentation models have enabled improved wound localization by capturing physiological changes associated with DFU [35], while advanced segmentation models have demonstrated strong performance in identifying multiple tissue types within wounds [36]. Additionally, granulation tissue detection models have been developed to support monitoring of wound healing progression using AI-based approaches [37]. Extending beyond static assessment, temporal machine learning frameworks have enabled longitudinal monitoring of wound healing trajectories across time [59], supporting more dynamic clinical evaluation. Furthermore, predictive models have been developed to estimate the risk of minor amputation in DFU patients using clinical variables [60], and machine learning approaches have also been applied to predict hard-to-heal ulcers and support prognosis assessment [61].

Overall, the included studies demonstrate a rapid expansion of AI applications in DFU assessment, encompassing wound segmentation, diagnostic classification, risk prediction, and longitudinal monitoring. The findings indicate that deep learning and machine learning approaches have significantly improved the accuracy and efficiency of DFU detection and evaluation, particularly through advanced imaging analysis and automated classification systems. However, several challenges remain, including variability in model generalizability across datasets, limited integration into clinical decision-making workflows, and the need for standardized validation using diverse and real-world data. These gaps highlight the necessity for future research to focus not only on model performance but also on clinical applicability, robustness, and implementation to ensure that AI-based DFU assessment tools can be effectively translated into routine clinical practice.

### Study Design

The study designs presented in these articles demonstrate various approaches and methodologies used to investigate DFU. Various research methods have been used in studies focusing on the detection, analysis, and management of DFU. One study used machine learning models such as random forest and support vector machine with Monte Carlo cross-validation to develop a DFU risk prediction model [54]. Deep learning methods were also applied in DFU image segmentation using Fast R-CNN and transfer learning to enhance wound detection accuracy [27]. Another study used a bilinear CNN to automatically measure wound depth and granulation tissue as a classification method based on medical images [28]. To evaluate the accuracy of DFU wound area measurements, a comparison was made between innovative applications, ruler methods, and ImageJ software to identify more efficient and accurate solutions [29]. The AHRF method was also developed to improve the accuracy of wound boundary segmentation under varying lighting conditions [30].

In a study evaluating AI recommendations for determining DFU amputation levels, an analysis was conducted on 60 patients, comparing AI recommendations with medical team decisions [66]. Another study used a SwishRes-U-Net deep learning model, consisting of a dual U-Net and a pretrained SwishResNet, for chronic wound segmentation, including DF [31]. To improve transparency in medical decision-making, the light gradient boosting machine (LightGBM) model, combined with SHAP, was used to develop a machine learning model capable of explaining DFU patient amputation predictions [53]. In real-time DFU wound classification studies, 2 CNN models, DFU_FNet and DFU_TFNet, were applied using hardware acceleration such as FPGA and GPU to enhance diagnostic efficiency [40]. In developing deep learning–based DFU detection platforms, the YOLOv5s model was used for wound segmentation, while other deep learning models were applied for infection and ischemia classification [22]. Moreover, explainable AI methods based on multi-CNN, combining DenseNet201, VGG19, and NASNetMobile, were applied to improve transparency in DFU diagnosis using algorithms like SHAP, LIME, and Grad-CAM [39].

The deep learning method ScoreDFUNet was developed for wound classification based on ulcers, infection, normal skin, and gangrene categories, aiming to enhance consistency in wound assessment [14]. For long-term DFU monitoring, the Siamese neural network method was applied to compare wound conditions over time to understand patient healing progression [56]. Additionally, CNN with superpixel color descriptor (SPCD) was used in research focused on identifying ischemia and infection in medical images to support early DFU diagnosis [55]. In clinical validation of AI-based applications for wound measurement and monitoring, studies were conducted comparing AI measurement results with manual methods performed by wound care nurses to assess the accuracy and reliability of the applications [17]. Finally, deep learning models were again developed using CNN approaches with hardware acceleration to enable more efficient real-time DFU wound classification [40].

The additional studies included in this review demonstrate diverse methodological designs, reflecting the evolving maturity of AI research in DFU assessment. Several studies used retrospective image datasets to train and evaluate deep learning models. A retrospective cohort study using a large dataset of DFU images was conducted to develop and validate segmentation and classification algorithms [57]. Similarly, an experimental model development study used publicly available DFUC (Diabetic Foot Ulcer Challenge) datasets to evaluate a hybrid deep learning classification model [67]. Another study used benchmark DFUC2020 and IEEE datasets to develop and evaluate an ensemble detection model for DFU localization [42]. In addition, a deep learning framework trained on publicly available Kaggle datasets was developed to assess classification performance and model generalization [41].

Prospective and clinical validation designs were also represented. A prospective clinical study evaluated healing trajectories using thermal and visual imaging to assess wound progression [58]. In addition, a comparative clinical validation study involving patients with various chronic wounds evaluated the reliability of an AI-enabled medical device for remote wound assessment [32].

Overall, these methodological approaches include experimental model development studies, retrospective dataset analyses, and prospective clinical validation studies, indicating a transition from purely technical model development toward clinically oriented evaluation of AI systems in DFU care.

### Key Findings

Building on the study characteristics described above, Table 4 summarizes each included study in terms of AI model type, dataset characteristics, application focus, and key outcomes.

**Table 4.** Synthesis analysis of 23 articles relevant to the objective.

	Studies	AI methods^a^	Datasets^b^	Key findings and summary
Wound segmentation, measurement, and characterization	[17,27-38]	Fast R-CNN; bi-CNN; DL mobile app; SwishRes-U-Net; wound viewer AI; AHRF; AI measurement app; CNN; explainable DL; CNN detection; thermal DL; benchmark DL; DL segmentation	DFU image datasets; DFU photos; multidevice images; in vitro and clinical wound patients; retrospective DFU cohort; DFUC2020 and IEEE datasets; clinical patients; DFU images; thermal DFU images; public datasets; multi-dataset DFU	AI demonstrates strong capability in automated wound detection, segmentation, and measurement, including depth and tissue characterization, with high accuracy and reliability comparable to clinical assessment.
Diagnostic classification and condition assessment	[14,39-52]	Hybrid DL classifier; XAI-FusionNet; ScoreDFUNet; ConMatFormer; DenseVAE-CL; few-shot DL; multimodal DL; attention DL; hybrid CNN; CNN+Transformer; efficient CNN; lightweight CNN; explainable DL; Transformer+XAI; CNN+ELM	DFU image datasets; DFUC2021 and Kaggle datasets; Kaggle DFU images; DFU images	Deep learning models achieve high accuracy in diagnostic classification and severity assessment, with explainable AI improving interpretability and clinical relevance.
Risk prediction, complication detection, and longitudinal monitoring	[22,53-65]	LightGBM; AutoML DL; DFUCare; DFU-Helper; CNN+SPCD; Siamese NN; Mask R-CNN fusion; ML; temporal ML; AI system; deep learning	Clinical datasets; DFU datasets; clinical images; serial DFU images; prospective clinical patients; clinical dataset; population dataset; longitudinal data; clinical images; DFU images	AI models effectively predict clinical outcomes, detect complications such as infection and ischemia, and enable longitudinal monitoring of wound healing with strong correlation to clinician assessment.
Clinical decision support and automated model development	[44,66-68]	AI-based DSS; AutoML DL; ML DSS; explainable DL	DFU clinical cases; DFU datasets; clinical dataset; DFU images	AI supports clinical decision-making with good agreement with physicians and enables automated predictive model development with high performance.

a AHRF: adaptive hybrid regression framework; AI: artificial intelligence; AutoML: automated machine learning; bi-CNN: bilinear convolutional neural network; CNN: convolutional neural network; DL: deep learning; DSS: decision support system; ELM: extreme learning machine; LightGBM: light gradient boosting machine; Mask R-CNN: mask region-based convolutional neural network; ML: machine learning; NN: neural network; R-CNN: region-based convolutional neural network; SPCD: superpixel color descriptor; XAI: explainable artificial intelligence.

b DFU: diabetic foot ulcer; DFUC: Diabetic Foot Ulcer Challenge.

#### Wound Segmentation, Measurement, and Characterization

A substantial body of evidence focused on the application of AI for wound segmentation, measurement, and characterization. Deep learning models such as Fast R-CNN demonstrated strong capability in detecting wound boundaries from medical images [27], while bilinear CNNs improved estimation of wound depth and granulation tissue compared with conventional approaches [28]. Probabilistic frameworks such as the AHRF enhanced segmentation accuracy under variable imaging conditions [30], and architectures such as SwishRes-U-Net further demonstrated robust performance across multiple datasets [31].

AI-powered medical devices showed strong reliability for remote wound assessment and tissue classification [32]. Validation studies of smart applications indicated that AI-based tools can provide efficient wound measurements with comparable accuracy to manual methods [29]. Additional evaluations demonstrated that AI-enabled measurement devices may reliably replace traditional measurement approaches in a clinical setting [17].

Deep learning–based diagnostic models achieved high accuracy in simultaneous wound segmentation and classification, thereby improving overall assessment efficiency [36]. Building on this, explainability-integrated models further enhanced segmentation performance while maintaining interpretable identification of wound regions, supporting clinical transparency [38]. In addition to structural segmentation, automated detection models demonstrated strong performance in identifying granulation tissue, enabling more objective evaluation of wound healing progression [37]. Furthermore, thermography-based segmentation frameworks improved detection accuracy by capturing temperature-related physiological changes associated with tissue damage [35]. Consistent with these advancements, benchmark evaluations demonstrated that segmentation models achieved competitive and reliable performance across standardized DFU datasets [33]. However, cross-dataset validation studies revealed reduced performance when applied to external datasets, highlighting ongoing limitations in model generalizability [34]. Overall, these findings indicate that AI-based segmentation and measurement technologies can improve objectivity and consistency in wound assessment.

#### Diagnostic Classification and Condition Assessment

Another major domain involved diagnostic classification and condition assessment. Ensemble frameworks combining multiple detection models demonstrated improved localization and diagnostic performance with high precision [42], while generative contrastive frameworks integrating DenseNet and variational autoencoders improved model generalization and classification accuracy across datasets [41].

Explainable AI approaches such as FusionNet enabled automated detection and analysis of DFU while providing interpretable outputs to support clinical interpretation [39]. The ScoreDFUNet model demonstrated strong capability in classifying DFU images into clinically relevant categories [14]. Real-time classification supported by hardware acceleration platforms further highlighted the feasibility of AI deployment in clinical environments [40].

Few-shot learning models achieved robust classification performance despite limited training data, demonstrating strong efficiency in data-scarce conditions [46]. Building on this, multimodal deep learning frameworks further improved classification accuracy by integrating complementary feature representations from multiple data sources [50]. In parallel, attention-based architectures achieved superior feature extraction, leading to improved classification accuracy compared with conventional models [47]. Similarly, hybrid feature extraction approaches combining texture descriptors with deep learning features enhanced classification performance [51]. Extending this approach, hybrid CNN–vision transformer models demonstrated superior performance by effectively capturing both local and global image features [45]. Moreover, efficient deep learning architectures achieved high classification accuracy while reducing computational complexity, supporting more practical implementation [43]. In line with this, lightweight architectures enabled accurate real-time DFU detection and grading with minimal computational burden [49]. Beyond performance, explainable AI frameworks improved diagnostic transparency by providing interpretable visual explanations without compromising accuracy [48]. Complementing these findings, transformer-based explainable models further enhanced interpretability while maintaining high classification performance suitable for clinical use [44]. Finally, hybrid CNN-ELM approaches achieved competitive classification performance while reducing training complexity, offering an efficient alternative for resource-limited settings [52]. Collectively, these findings indicate increasing maturity of AI-based diagnostic classification systems for DFU assessment.

#### Risk Prediction, Complication Detection, and Longitudinal Monitoring

Several studies focused on risk prediction, complication detection, and longitudinal monitoring of DFU. Machine learning models demonstrated the ability to estimate clinical risk such as amputation likelihood and disease progression, with moderate-to-high discriminatory performance across metrics including accuracy, sensitivity, specificity, and area under the curve [54]. However, evidence of external validation remained limited, with most studies relying on internal testing datasets [53]. Severity classification frameworks integrating imaging and predictive modeling also demonstrated high accuracy in predicting ulcer severity and supporting clinical risk stratification [57].

AI-based monitoring approaches further supported longitudinal assessment of wound progression. The DFUCare model enabled automated detection and monitoring of wound healing and complications such as infection and ischemia [22], while CNN approaches incorporating SPCDs demonstrated the capability in detecting ischemia and infection [55]. Siamese neural network frameworks enabled comparison of wound conditions over time to track healing trajectories [56], and image fusion models integrating thermal and visual imaging showed strong agreement with clinician measurements in monitoring healing progression [58].

Machine learning models achieved high predictive accuracy in estimating minor amputation risk, thereby supporting more informed clinical decision-making [60]. Building on this, predictive models demonstrated strong discriminative ability in identifying hard-to-heal DFUs at early stages, enabling earlier intervention strategies [61]. Furthermore, externally validated models achieved reliable performance in predicting DFU infection across different clinical settings, indicating good generalizability [62]. At the population level, predictive models also achieved strong accuracy in identifying individuals at risk of developing DFU, supporting preventive care approaches [63].

In addition to risk prediction, temporal machine learning frameworks achieved high accuracy in predicting wound healing trajectories across multiple clinical visits, allowing for dynamic monitoring of patient outcomes [59]. Consistent with this, AI-based monitoring systems demonstrated strong agreement with clinical assessments in evaluating wound severity and progression, reinforcing their clinical applicability [64]. Moreover, thermography-based deep learning models improved early detection sensitivity for DFU-related complications by capturing physiological changes, further enhancing early diagnostic capability [65]. These findings highlight the potential role of AI in supporting early risk identification and objective monitoring of DFU progression.

#### Clinical Decision Support and Automated Model Development

**Figure 2.**
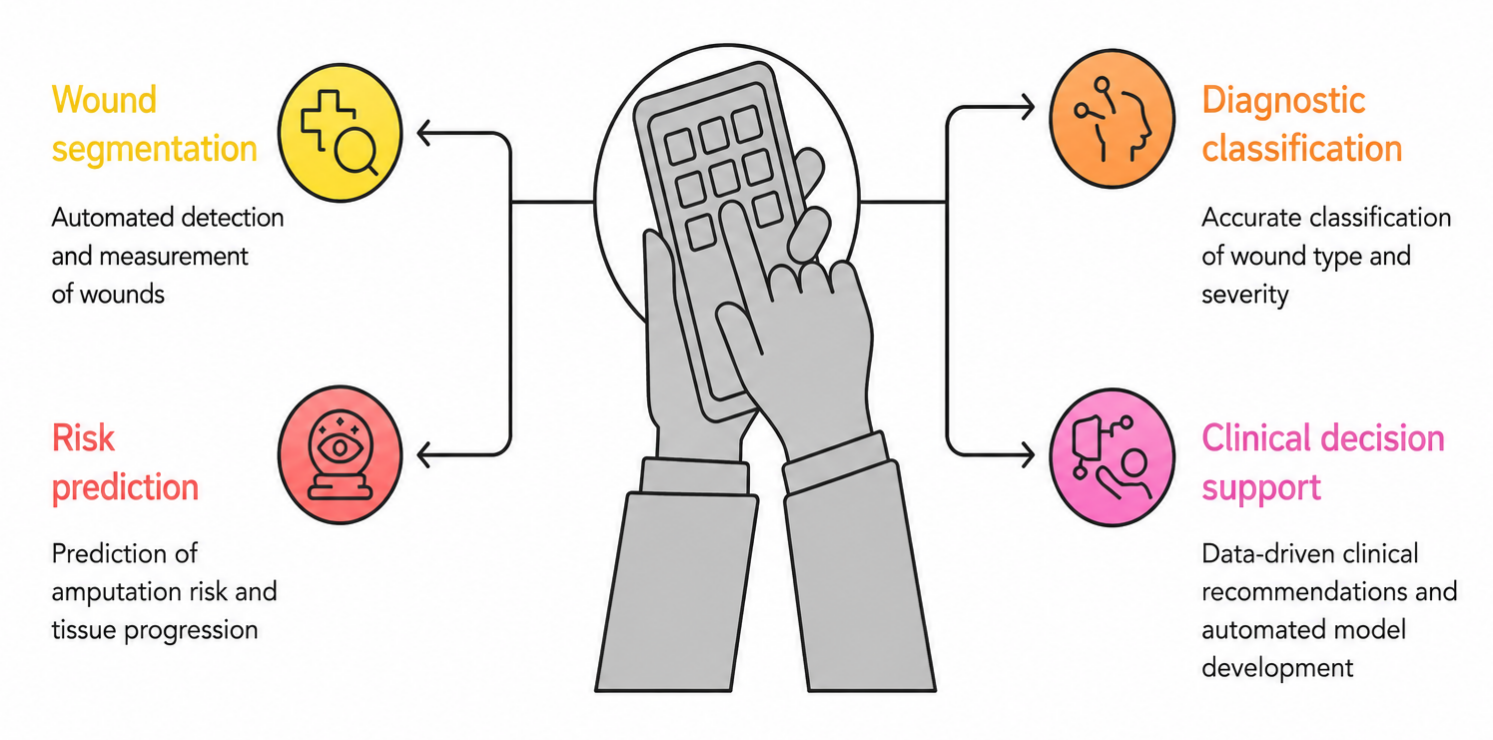
Key benefits of artificial intelligence in diabetic foot ulcer assessment. AI: artificial intelligence.

AI has also been applied to support clinical decision-making and automated model development. Studies reported that AI-generated recommendations for amputation level were generally consistent with physician judgment, indicating potential for decision support in clinical practice [66]. Explainability and transparency remain important components of decision support systems. Hybrid deep learning frameworks incorporating explainable AI methods improved interpretability of predictions and supported clinician understanding of decision pathways [67]. Machine learning–based clinical decision models achieved high predictive performance in supporting risk stratification and treatment planning, thereby enhancing the precision of clinical decision-making in DFU management [68]. Building on this, explainability-driven deep learning approaches further improved model transparency while maintaining strong diagnostic performance, thereby increasing clinician trust and facilitating the integration of AI-based decision systems into clinical practice [44]. Overall, these findings suggest that AI-based decision support tools may enhance clinical workflow efficiency and support evidence-based DFU management. The specific AI approaches and their clinical application domains were mapped to provide an overview of how AI has been used to support DFU assessment (Figure 2).

## Discussion

### Principal Findings

#### Wound Segmentation, Measurement, and Characterization

Deep learning models have increasingly been applied to the detection and segmentation of DFU, offering a more standardized and reproducible alternative to conventional manual assessment. These models enable automated boundary delineation, depth classification, and tissue identification, which are key elements in DFU management [29,30].

The growing use of real-time and hardware-accelerated models such as DFU_TFNet further indicates the potential for deployment in point-of-care environments [54]. Performance is increasingly evaluated using standardized segmentation metrics such as Dice score and intersection over union (IoU), allowing comparison across architectures including SwishRes-U-Net, SegNet, and U-Net [55].

Recent studies also demonstrate continued progress in AI-enabled wound measurement technologies. An imaging-based AI system integrating advanced sensing technologies such as LiDAR (light detection and ranging) demonstrated high accuracy in quantifying wound dimensions and tissue composition, supporting its potential role as an objective assessment tool [57]. An AI-powered wound imaging device validated in clinical settings demonstrated strong agreement with clinician measurements, reinforcing its reliability for both in-person and remote wound assessment [67].

Deep learning–based diagnostic models achieved high accuracy in simultaneous wound segmentation and classification, indicating strong potential to improve efficiency and objectivity in DFU assessment, although their reliance on curated datasets may limit real-world applicability [36]. Building on this, explainability-integrated models not only improved segmentation performance but also enhanced interpretability, which is essential for clinical adoption, yet their usefulness depends on alignment with clinician reasoning processes [38]. In parallel, automated detection models demonstrated strong performance in identifying granulation tissue, supporting more objective evaluation of healing progression, although variability in wound characteristics across patients may affect consistency [37]. Furthermore, thermography-based segmentation frameworks improved detection accuracy by capturing physiological changes, but their dependence on specialized imaging modalities may limit scalability in resource-constrained settings [35]. While benchmark evaluations confirmed consistent performance across standardized datasets, these controlled environments may not fully reflect the heterogeneity of real-world clinical data [33]. This limitation is further reinforced by cross-dataset validation studies showing reduced performance on external datasets, highlighting persistent challenges in generalizability and the need for more diverse training data [34].

These advances suggest that AI-based measurement tools may help reduce subjectivity and improve consistency in DFU evaluation, although integration into routine workflows remains a key challenge. However, despite the strong technical performance reported in these studies, broader implementation will require additional validation across diverse patient populations and integration testing within clinical workflows. Future research should focus on prospective validation, clinician feedback, and interoperability with electronic health records to ensure that AI-based DFU tools are not only accurate but also seamlessly embedded into routine wound care practices.

#### Diagnostic Classification and Condition Assessment

Recent advances in deep learning architectures further demonstrate improvements in diagnostic performance and model robustness. A hybrid deep learning architecture integrating convolutional networks, attention mechanisms, and transformer modules has shown high classification accuracy while improving interpretability through explainable AI techniques, suggesting enhanced capability for complex feature extraction [58]. An ensemble detection framework combining multiple object detection models demonstrated improved localization performance, supporting its potential use in automated screening [14]. In addition, a generative contrastive deep learning framework achieved very high classification accuracy and improved generalization across datasets, indicating continued methodological innovation in DFU image analysis [32]. Despite these developments, most studies primarily report technical performance without examining usability or integration into routine workflows. External validation across diverse patient populations also remains limited, highlighting the need for further prospective research before widespread clinical adoption. Despite these advances, most studies remain focused on technical validation, highlighting the ongoing need for external validation and real-world clinical evaluation.

Few-shot learning models achieved robust classification performance despite limited training data, suggesting strong potential for application in data-scarce environments, although their stability across heterogeneous clinical images remains uncertain [46]. Extending this, multimodal deep learning frameworks significantly improved classification accuracy by integrating complementary feature representations, yet the resulting increase in model complexity may hinder real-time clinical deployment [50]. Similarly, attention-based architectures enhanced feature extraction and classification performance, although issues related to interpretability and reproducibility across datasets remain insufficiently addressed [47]. Hybrid feature extraction approaches further improved classification accuracy by combining handcrafted and deep features, but their adaptability to new datasets may be limited [51]. In addition, CNN–vision transformer models achieved superior performance by capturing both local and global features, although their computational demands may restrict implementation in low-resource settings [45]. To address these constraints, efficient deep learning architectures achieved high classification accuracy while reducing computational complexity, suggesting improved feasibility for broader implementation [43]. Complementing this, lightweight models enabled accurate real-time DFU detection and grading, although potential trade-offs in handling complex cases require further investigation [49]. Beyond performance, explainable AI frameworks improved diagnostic transparency through interpretable visual outputs, yet their clinical relevance depends on clinician acceptance and understanding [48]. This trend is further supported by transformer-based explainable models, which enhance interpretability while maintaining high performance, although standardized evaluation of explainability remains lacking [44]. Finally, hybrid CNN-ELM approaches achieved competitive performance with reduced training complexity, offering efficiency advantages, but their scalability across larger and more diverse datasets remains uncertain [52].

#### Risk Prediction, Complication Detection, and Longitudinal Monitoring

Recent developments in AI-based models for DFU detection and risk prediction highlight their potential to support clinical decision-making and patient monitoring. Automated machine learning approaches have demonstrated promising performance compared with traditional methods, particularly for localized DFU risk assessment and real-time monitoring [27]. These approaches may offer more objective and consistent evaluations, which are critical for early intervention and personalized care planning. Additional evidence from recent studies further supports the potential role of AI-based classification models in contributing to clinical risk stratification. An AI-enhanced imaging framework combining segmentation and classification demonstrated strong performance in predicting ulcer severity based on Wagner grading, suggesting that image-based classification models may serve as indirect indicators of clinical risk and support early intervention strategies [42]. However, similar to earlier findings, these approaches primarily rely on retrospective datasets and require further prospective validation before being applied to recurrence prediction or long-term risk modeling.

Despite these promising results, broader implementation requires further validation and user-centered design improvements. Most studies focus on technical performance without addressing practical usability or integration into routine clinical workflows. Additionally, while internal validation shows strong metrics, external validation across diverse populations remains limited. Future work should prioritize prospective testing, compatibility with electronic health systems, and feedback from end users to ensure that AI tools are not only accurate but also clinically meaningful and scalable.

AI-based tools such as DFUCare, SPCD-based CNN models, and Siamese neural networks have been applied to support the monitoring of DFU progression and detection of complications including infection and ischemia [22,31]. These approaches may reduce variability associated with conventional visual assessment and support more consistent decision-making in complex DFU cases. Some studies also reported agreement between AI-generated recommendations and clinician judgment in amputation planning [56], suggesting a potential role for AI as a decision-support tool.

Emerging evidence also highlights the expanding role of AI in supporting longitudinal monitoring and remote management of DFU. An image analysis model integrating thermal and visual imaging demonstrated strong agreement with clinician assessments when evaluating wound healing trajectories, suggesting its potential to provide objective monitoring of wound progression [41]. An AI-enabled medical device designed for remote wound assessment showed high reliability in measuring wound parameters and classifying tissue characteristics, reinforcing its potential to support telemedicine and reduce variability in clinical assessment [67]. These findings suggest a gradual shift from algorithm development toward clinically applicable monitoring tools, although broader implementation will require further validation across diverse clinical settings.

Machine learning models achieved high predictive accuracy in estimating minor amputation risk, highlighting their potential to support clinical decision-making, although reliance on retrospective datasets may limit predictive reliability in prospective settings [60]. Building on this, predictive models for hard-to-heal DFUs demonstrated strong discriminative ability, enabling earlier identification of patients at risk of poor outcomes, yet their generalizability across populations with different comorbidities remains uncertain [61]. Furthermore, externally validated models achieved reliable performance in predicting DFU infection, suggesting improved robustness, although broader validation across diverse health care systems is still required [62]. At a population level, predictive models achieved strong accuracy in identifying individuals at risk of developing DFU, supporting preventive strategies, but their integration into routine screening programs remains underexplored [63].

In addition to static risk prediction, temporal machine learning frameworks achieved high accuracy in predicting wound healing trajectories across clinical visits, enabling dynamic monitoring of patient outcomes, although their dependence on longitudinal data may limit use in settings with irregular follow-up [59]. Consistent with this, AI-based monitoring systems demonstrated strong agreement with clinical assessments in evaluating wound severity and progression, supporting their clinical applicability, yet their impact on workflow efficiency and patient outcomes remains insufficiently studied [64]. Moreover, thermography-based deep learning models improved early detection sensitivity for DFU-related complications, suggesting added diagnostic value, although limited access to thermal imaging may constrain widespread implementation [65]. Usability testing and workflow integration were rarely reported, highlighting the need for prospective, user-centered validation in clinical environments.

#### Clinical Decision Support and Automated Model Development

Mobile-based AI applications such as the Diabetic Foot Smart APP and eKare Insight have demonstrated potential to support more objective and standardized DFU measurement in both clinical and remote care settings [40,53]. These tools may help reduce interobserver variability and facilitate longitudinal documentation of wound-healing progress, addressing one of the main limitations of conventional visual assessment methods [17]. In addition, the use of automated image-based analysis enables clinicians to obtain more consistent wound measurements without requiring specialized equipment, which may be particularly beneficial in settings with limited access to wound-care expertise.

From an implementation perspective, mobile-based AI platforms align well with telemedicine and mobile health strategies, allowing wound assessment and monitoring to occur closer to the patient and reducing dependence on in-person visits [40,53]. Their compatibility with widely available smartphone devices increases accessibility and offers opportunities to support community-based care and remote clinical supervision.

Explainable AI approaches such as FusionNet aim to improve transparency in AI-supported DFU assessment by providing visual explanations of classifier outputs using techniques such as SHAP, LIME, or Grad-CAM [66]. These tools may help clinicians interpret AI-generated assessments alongside clinical indicators such as infection and ischemia status. Some studies reported comparable diagnostic performance between AI systems and clinicians [39]; however, validation remains limited and further evaluation is required across diverse clinical settings.

These findings highlight the growing role of explainable AI in enhancing clinical utility and decision-making alignment between AI systems and medical experts. Notably, FusionNet has demonstrated performance that surpasses junior and intermediate dermatologists while aligning closely with senior dermatologists, suggesting that AI can serve as a valuable support tool in DFU assessment [39]. This level of agreement is particularly relevant in critical care scenarios where early detection and accurate severity classification are essential for preventing complications such as amputation.

Moreover, the adoption of hardware-accelerated AI models like DFU_TFNet further enhances the practicality of these tools in real-time clinical environments [54]. By combining speed and precision, such models offer the potential for immediate deployment in primary care or telemedicine settings, especially in cases requiring rapid wound classification and triage.

The integration of explainable AI components within hybrid deep learning models further emphasizes the importance of transparency in AI-supported DFU assessment. A hybrid deep learning classification model incorporating attention mechanisms and interpretability frameworks provides visualization of decision pathways, which may enhance clinician understanding and trust in AI-generated outputs [58]. This growing emphasis on explainability reflects an important step toward improving clinical acceptability and facilitating integration into routine practice, although standardized evaluation of interpretability remains limited.

Despite these advantages, broader clinical deployment remains limited. Most studies primarily report technical feasibility and reliability, with relatively little evidence on real-world integration into existing clinical workflows, interoperability with electronic health information systems, or clinician and patient acceptability [17,40].

Machine learning–based clinical decision models achieved high predictive performance in supporting risk stratification and treatment planning, indicating strong potential to augment clinical decision-making, although their effectiveness ultimately depends on integration within existing clinical workflows [68]. Building on this, explainability-driven deep learning approaches improved transparency and clinician trust in AI systems, which is critical for adoption, yet the absence of standardized evaluation frameworks for interpretability remains a key barrier to widespread clinical implementation [44]. Furthermore, standardized evaluation frameworks and prospective multisite validation are still required to determine the generalizability, safety, and long-term clinical value of these applications. Accordingly, the implementation of mobile AI tools for DFU assessment should continue to be approached as a supportive adjunct to clinical judgment rather than a replacement for comprehensive wound evaluation.

### Comparison With Prior Work

This review extends prior work in wound assessment by focusing specifically on AI-based approaches for DFU evaluation. While earlier reviews emphasized manual or standardized clinical assessment tools, the present review highlights a shift toward automated systems designed to support diagnostic consistency and wound monitoring. AI-enabled platforms such as eKare Insight, FusionNet, and DFUCare illustrate this transition toward data-driven assessment and integration with digital health technologies.

Unlike traditional wound-care approaches, AI systems have the potential to reduce observer-dependent variability and support longitudinal tracking; however, translation into routine clinical practice remains limited by challenges in validation, usability, and workflow integration. This review therefore contributes by mapping current AI approaches, summarizing reported outcomes, and identifying key implementation gaps for future research.

### Research Limitation

Lastly, although some AI tools reported agreement with clinician decision-making, only a small number of studies provided detailed statistical validation or external testing across different health care environments. As a result, the true robustness and reproducibility of these systems in everyday clinical practice remain uncertain. Many models were evaluated under controlled research conditions, which may not fully reflect the variability in patient characteristics, image quality, and workflow demands encountered in real-world settings. Future studies should therefore prioritize multicenter validation, standardized reporting, and prospective clinical evaluation to better determine the safety, reliability, and clinical readiness of AI-based DFU assessment tools. This highlights the need for rigorous, externally validated, and prospectively designed studies to establish clinical robustness and implementation feasibility.

### Future Direction

Future research should prioritize the translation of AI-based DFU assessment tools into real-world clinical environments through prospective, multicenter evaluation. This includes external validation across diverse populations, imaging conditions, and care settings to improve generalizability and assess robustness beyond controlled research contexts. In addition, greater emphasis is needed on usability testing and user-centered design to ensure that AI systems align with clinician workflows, minimize cognitive burden, and enhance rather than replace clinical judgment.

Integration with digital health infrastructures such as electronic health records and telemedicine platforms represents another important direction to support continuity of care, remote monitoring, and multidisciplinary wound management. Explainable AI approaches also warrant further investigation to improve transparency and clinician trust, particularly in high-risk decision contexts. Finally, future work should adopt standardized reporting frameworks and evaluation metrics to enable consistent comparison across studies and facilitate the safe, scalable, and ethical implementation of AI-supported DFU assessment in clinical practice.

### Ethical and Regulatory Implications of AI-Based DFU Assessment

The clinical application of AI-based DFU assessment tools must also be viewed through an ethical and regulatory lens. Key issues include transparency in algorithm development and decision pathways, fairness in performance across different patient groups, and protection of personal health data. AI models trained using limited or homogeneous datasets may introduce algorithmic bias, potentially resulting in unequal performance across demographic or clinical subgroups. Similarly, the use of clinical wound photographs raises important data governance and privacy concerns, particularly in telemedicine and mobile health environments. Ensuring model explainability, secure data handling, and appropriate regulatory oversight will therefore be essential to support clinician trust, safeguard patient rights, and promote equitable AI implementation in DFU care. Future research should incorporate ethical risk assessment alongside technical validation to support the responsible deployment of AI-enabled wound assessment systems.

### Conclusions

The integration of AI into DFU assessment and management demonstrates promising technical and methodological advancements in areas such as diagnostic accuracy, risk prediction, wound monitoring, and clinical decision support. From machine learning to deep learning and explainable AI, current technologies offer innovative approaches that may reduce subjectivity and support early detection, particularly in resource-limited settings. However, the transition from experimental performance to routine clinical implementation remains hindered by challenges such as limited dataset diversity, lack of external validation, and insufficient integration into clinical workflows. To bridge this gap, future research should prioritize multicenter validation, user-centered design, interoperability, and alignment with clinical standards. Through such efforts, AI-based tools may become reliable and scalable components of DFU assessment in clinical practice.

## Supplementary material

10.2196/77925 Multimedia Appendix 1 Summary of literature search strategy: databases, keywords, and selection results.

10.2196/77925 Checklist 1 PRISMA-ScR checklist.

## Abbreviations

AHRF: associative hierarchical random field
AI: artificial intelligence
CNN: convolutional neural network
DEPA: depth, extent of bacterial colonization, phase of healing, and associated aetiology
DFU: diabetic foot ulcer
DFUAS: Diabetic Foot Ulcer Assessment Scale
DFUC: Diabetic Foot Ulcer Challenge
DMIST: depth, maceration, inflammation/infection, size, tissue type, type of wound edge, tunneling
ELM: extreme learning machine
FPGA: field-programmable gate array
GPU: graphics processing unit
Grad-CAM: gradient-weighted class activation mapping
IoU: intersection over union
LiDAR: light detection and ranging
LightGBM: light gradient boosting machine
LIME: local interpretable model-agnostic explanations
PCC: population, concept, context
PRISMA-ScR: Preferred Reporting Items for Systematic Reviews and Meta-Analyses Extension for Scoping Reviews
SHAP: Shapley Additive Explanations
SPCD: superpixel color descriptor
