# Supplementary material for: AI-Based Models for Diabetic Foot Ulcer Assessment: Scoping Review
Source: JMIR Diabetes. 2026 Jul 8;11:e77925. doi: 10.2196/77925 (PMC13345344; doi:10.2196/77925)
Supplement: Multimedia Appendix 1 [file diabetes-v11-e77925-s001.docx]

| **Database** | **Keywords** | **Initial Articles** | **Final Articles** | **Date Accessed** |
| --- | --- | --- | --- | --- |
| PubMed | ((("Diabetic Foot"[MeSH] OR "Diabetic Foot Ulcer"[MeSH] OR "diabetic foot ulcer"[Title/Abstract] OR "diabetic foot"[Title/Abstract] OR "foot ulcer"[Title/Abstract] OR "diabetic wound"[Title/Abstract] OR DFU[Title/Abstract]) AND (english[Filter])) AND (("Artificial Intelligence"[MeSH] OR "artificial intelligence"[Title/Abstract] OR "machine learning"[Title/Abstract] OR "deep learning"[Title/Abstract] OR "neural network*"[Title/Abstract] OR "computer vision"[Title/Abstract] OR ChatGPT[Title/Abstract]) AND (english[Filter]))) AND (("Wound Healing"[MeSH] OR "assessment"[Title/Abstract] OR "evaluation"[Title/Abstract] OR "measurement"[Title/Abstract] OR "detection"[Title/Abstract] OR "classification"[Title/Abstract] OR "segmentation"[Title/Abstract] OR "diagnosis"[Title/Abstract]) AND (english[Filter])) | 253 | 245 | April, 2026 |
| Proquest | abstract(("diabetic foot ulcer" OR "diabetic foot" OR "foot ulcer" OR "diabetic wound" OR DFU)) AND abstract(("artificial intelligence" OR "machine learning" OR "deep learning" OR "neural network" OR ChatGPT)) AND abstract(("assessment" OR "evaluation" OR "measurement" OR "detection" OR "classification" OR "segmentation" OR "diagnosis")) | 134 | 123 | April, 2026 |
| Scopus | ABS("diabetic foot ulcer" OR "diabetic foot" OR "foot ulcer" OR "diabetic wound" OR DFU) AND ABS("artificial intelligence" OR "machine learning" OR "deep learning" OR "neural network" OR "computer vision" OR ChatGPT) AND ABS("assessment" OR "evaluation" OR "measurement" OR "detection" OR "classification" OR "segmentation" OR "diagnosis") | 648 | 286 | April, 2026 |
