# Supplementary material for: AI-Based Models for Diabetic Foot Ulcer Assessment: Scoping Review
Source: JMIR Diabetes. 2026 Jul 8;11:e77925. doi: 10.2196/77925 (PMC13345344; doi:10.2196/77925)
Supplement: Checklist 1 [file diabetes-v11-e77925-s002.docx]

**AI-Based Models for Diabetic Foot Ulcer Assessment: Scoping Review**

Preferred Reporting Items for Systematic Reviews and Meta-Analyses Extension for Scoping Reviews (PRISMA-ScR) Checklist

| **SECTION** | **PRISMA-ScR ITEM** | **NO.** | **REPORTING LOCATION / DETAILS** | **REPORTED ON PAGE #** |
| --- | --- | --- | --- | --- |
| **TITLE** | | | | |
|  | Title | 1 | The article title identifies the report as a scoping review: “AI-Based Models for Diabetic Foot Ulcer Assessment: Scoping Review.” | Page 1 |
| **ABSTRACT** | | | | |
|  | Structured summary | 2 | A structured abstract is provided with Background, Objective, Methods, Results, and Conclusions. It summarizes the review purpose, databases, eligibility framework, data charting, narrative synthesis, number of included studies (46), main AI application domains, and key gaps. | Page 1 |
| **INTRODUCTION** | | | | |
|  | Rationale | 3 | The rationale describes the clinical burden of diabetic foot ulcers, limitations of conventional wound assessment, and the need to map AI-based approaches for DFU assessment, including segmentation, classification, monitoring, and decision support. | Pages 1-2 |
|  | Objectives | 4 | The review question is explicitly stated: how AI-based models have been developed and applied for DFU assessment, and what their methodological characteristics and reported performance outcomes are. The objective is to map and synthesize evidence on AI-based models for DFU assessment. | Page 2 |
| **METHODS** | | | | |
|  | Protocol and registration | 5 | The manuscript states that the review was not prospectively registered in any database. No separate protocol registration is reported. | Page 3 |
|  | Eligibility criteria | 6 | Eligibility criteria are described using the PCC framework. Population: individuals with DFU. Concept: AI-based models including machine learning, deep learning, and computer vision for DFU assessment. Context: clinical, experimental, or health care-related technological settings. Peer-reviewed, English full-text articles published between 2014 and 2026 were included. | Pages 2-3 |
|  | Information sources | 7 | Information sources are reported as PubMed, ProQuest, and Scopus. The manuscript also states that full-text access was sought through institutional and other available sources when needed. | Pages 2-3 |
|  | Search | 8 | The manuscript reports that database-specific search strings were used with terms related to diabetic foot ulcers, artificial intelligence, machine learning, deep learning, and wound assessment. Full search strings are provided in Multimedia Appendix 1. | Page 2; Multimedia Appendix 1 |
|  | Selection of sources of evidence | 9 | Records were imported into Rayyan, duplicates were removed, titles and abstracts were independently screened by two reviewers, potentially eligible studies underwent independent full-text assessment, and disagreements were resolved through discussion or third-reviewer consultation. | Page 3 |
|  | Data charting process | 10 | Data extraction/charting procedures are described. Extracted data included study characteristics and AI application information. Two reviewers independently categorized studies into predefined domains, with disagreements resolved by discussion or third-reviewer adjudication. | Page 3 |
|  | Data items | 11 | Data items included author and year, country, study design, dataset characteristics, AI methods, application domain, and reported performance metrics, as well as information on AI-based DFU assessment models. | Page 3 |
|  | Critical appraisal of individual sources of evidence | 12 | No formal risk-of-bias or critical appraisal assessment was conducted, consistent with the scoping review methodology. | Page 3 |
|  | Synthesis of results | 13 | The extracted data were synthesized narratively using a deductive thematic approach. Studies were grouped into predefined AI application domains: segmentation/measurement, diagnostic classification, risk prediction/monitoring, and clinical decision support. | Page 3 |
| **RESULTS** | | | | |
|  | Selection of sources of evidence | 14 | The selection process is reported in text and Figure 1: 654 records identified, 310 duplicates removed, 344 records screened, 196 excluded, 148 reports sought for retrieval, 41 not retrieved, 107 full-text articles assessed, 61 excluded, and 46 studies included. | Pages 3-4; Figure 1 |
|  | Characteristics of sources of evidence | 15 | Characteristics of included studies are summarized in Table 1 and the Study Characteristics section, including AI methods, application domains, data sources, and primary outcomes. Domain distribution is reported in Table 2 and the evidence gap map is presented in Table 3. | Pages 3-5; Tables 1-3 |
|  | Critical appraisal within sources of evidence | 16 | Not applicable. No formal critical appraisal or risk-of-bias assessment was conducted for individual sources of evidence. | Page 3 |
|  | Results of individual sources of evidence | 17 | Results from the included sources are charted and summarized by AI application domain in Table 1 and Table 4, including AI methods, datasets/data sources, primary outcomes, and key findings. | Pages 4-9; Tables 1 and 4 |
|  | Synthesis of results | 18 | The synthesis is reported narratively across the four domains: wound segmentation/measurement/characterization; diagnostic classification/condition assessment; risk prediction/complication detection/longitudinal monitoring; and clinical decision support/automated model development. | Pages 5-9 |
| **DISCUSSION** | | | | |
|  | Summary of evidence | 19 | The Discussion summarizes the main findings and links them to the review objective, emphasizing the promise of AI for DFU segmentation, classification, monitoring, and decision support, while highlighting gaps in validation, generalizability, workflow integration, and implementation. | Pages 10-13 |
|  | Limitations | 20 | Limitations are discussed, including limited detailed statistical validation and external testing, uncertainty regarding robustness and reproducibility in everyday clinical practice, and the need for multicenter validation, standardized reporting, and prospective clinical evaluation. | Page 13 |
|  | Conclusions | 21 | The conclusions interpret the evidence in relation to AI-based DFU assessment and emphasize the need for multicenter validation, user-centered design, interoperability, alignment with clinical standards, and translation into routine clinical practice. | Page 13 |
| **FUNDING** | | | | |
|  | Funding | 22 | The funding statement reports support from the Thematic Research Group (TRG) Batch 1, Hasanuddin University, and states that the funding body had no role in study design, data collection, analysis, interpretation, or manuscript writing. | Page 14 |

Abbreviations: AI: artificial intelligence; DFU: diabetic foot ulcer; PCC: population, concept, context; PRISMA-ScR: Preferred Reporting Items for Systematic Reviews and Meta-Analyses Extension for Scoping Reviews.

Source checklist: Tricco AC, Lillie E, Zarin W, O'Brien KK, Colquhoun H, Levac D, et al. PRISMA Extension for Scoping Reviews (PRISMA-ScR): Checklist and Explanation. Ann Intern Med. 2018;169:467-473. doi: 10.7326/M18-0850.
